# Empathy AI in healthcare

**DOI:** 10.3389/fpsyg.2025.1680552

**Published:** 2025-12-17

**Authors:** Karishma Muthukumar

**Affiliations:** University of Cambridge, Cambridge, United Kingdom

**Keywords:** Empathy, human-machine interaction, AI in healthcare, AI in medicine, conversational AI, empathic AI, chatbot evaluation

## Abstract

**Introduction:**

AI is changing healthcare and potentially even how humans interpret and express empathy. Patients and healthcare professionals are consulting AI for medical concerns, so it is critical to identify when AI expressions of empathy are helpful versus harmful. Whether or not AI is considered genuinely empathetic, the common goal is to improve AI outputs as well as healthcare outcomes. The paper explores how generative AI can impact care in a digital future.

**Methods:**

We develop a tool for evaluating empathy called the Chatbot Compassion Quotient, or CCQ. We created a set of nine prompts, assessing compassion in various capacities, including delivering difficult news and alleviating frustration, based on the psychology literature. We compare ChatGPT and Claude-generated responses with responses from healthcare professionals. Participants also guessed which of the responses was AI-generated versus human-generated. In this corollary to the Turing test, the central question “can machines think?” became “can machines demonstrate compassion?” Thirty participants rated 3 responses to 9 scenarios on a 5-point Likert scale of 1 (not at all compassionate) to 5 (very compassionate). Responses corresponded to either ChatGPT, human, or Claude-generated results and were labeled A, B, and C in random order. After rating on the compassion scale, participants were asked to identify which, between two options, was AI-generated.

**Results:**

Results indicated that participants considered responses from ChatGPT (aggregate score: 4.1 out of 5) and Claude (aggregate score: 4.1 out of 5) more empathetic than human (aggregate score: 2.6 out of 5) responses, with length being a potential factor impacting evaluations. Longer responses were typically rated as more compassionate. The scores for ChatGPT and Claude were comparable. Responses that appeared most obviously AI-generated performed well compared to human responses. High-scoring responses were action-oriented with multiple forms of social support.

**Conclusion:**

The study highlights the promise of human-machine synergy in healthcare. AI may alleviate fatigue and burnout in the medical field, contributing thorough responses that offer insight into patient-centered care. Further research can build on these preliminary findings to evaluate and improve expressions of empathy in AI.

## Introduction

Computational technologies are changing healthcare practices and potentially even how humans interpret empathy. The motivation for this research is to evaluate whether and, if applicable, to what extent AI can show elements of empathy. Groundbreaking advancements in cognitive sciences are motivating more people to study traces of empathy in AI. AI can, in some cases, be less biased than humans and help people feel understood (Inzlicht et al., 2023). While some may consider AI never genuinely empathetic, the broader aim is to know when AI expressions of empathy are productive versus harmful (Inzlicht et al., 2023).

Individual people have different ideas on what is empathetic. Differences in perspective make defining empathy a problematic task. Empathy in healthcare is becoming a more significant focus, with related metrics like quality of care, patient-centered care, and patient satisfaction as core objectives. The paper explores whether chatbots can help bridge the gap in defining and offering empathy and reimagining care for a digital future.

Empathy is a central focus in society, specifically in healthcare, and is strongly associated with rationality, burnout, interpersonal skills, and social behaviors (Hall and Schwartz, 2022). Definitions list several aspects and present empathy as a multidimensional concept. However, descriptions of empathy are not universal, as measurements differ. Social psychology highlights internal responses, including emotions and the inclination to help, while medical applications focus on the caregiver’s actions (Hall et al., 2024). Clinical empathy is understanding the patient’s perspective, communicating an understanding, and acting on it (Mercer and Reynolds, 2002). It is about understanding suffering and being motivated to alleviate pain. A review of clinician behaviors identified elements such as self-disclosure, conversation, clarity, respect, sensitivity, expression, exploration, and shared decision-making (Lelorain et al., 2012). For example, clinicians practice empathy through reassurance and silence, creating space for the patient’s emotions (Westendorp, 2021).

## Context

### Measuring empathy in healthcare

Healthcare research in empathy may not generalize across settings when interventions and measures are not specified (Hall et al., 2024). Researchers must agree on a definition of empathy before studying its applications. Different components of empathy may have unique interactions. For instance, emotion-sharing was associated with greater burnout, whereas cognitive empathy and compassion were associated with lower burnout (Hall et al., 2024). Researchers can define and explore relevant variables depending on what the intervention intends to address. Various measurement tools can help measure empathy in healthcare.

The Medical Interview Satisfaction Scale is a tool developed to measure patient satisfaction levels based on physician behaviors (Wolf et al., 2017). It encompasses cognitive, affective, and behavioral components assessed with statements (e.g., “I really felt understood by my doctor”) that patients rate for agreement on a scale of 1 to 5. Similarly, the Consultation and Relational Empathy (CARE) scale aims to measure elements of empathy through phrasing that reflects a patient-centered approach. Patients rate how often a healthcare provider has performed ten behaviors, including “fully understanding concerns, “being interested in you as a whole person,” and “letting you tell your story” (Mercer, 2004).

Evaluating empathy in clinical settings requires other important contextual considerations. For instance, individuals must assess whether the emotional response is appropriate. Intense responses are empathetic when considered in a proper context, given that a minor bruise is unlike a diagnosis of a terminal illness (Baron-Cohen and Wheelwright, 2004). The same response can be empathetic in one scenario and inappropriate in another. Additionally, empathy can look different across various cultures. Empathy is universal; however, cultural norms may affect how it is expressed or experienced. Furthermore, empathy and social skills, or awareness of social norms, may be confounded.

### Large language models (LLMs) in healthcare

Large language models like Chat-GPT are reshaping healthcare delivery, presenting a growing responsibility to reconfigure tools to measure the impact on empathy. Individuals, including patients and healthcare professionals, interact with Chat-GPT for a variety of healthcare scenarios. Given the increasing use and integration of patient information, several institutions have developed institution-specific tools.

A recent study within a healthcare context evaluated general LLMs in terms of quality and empathy. Chat-GPT responses to medical symptoms posted on a discussion board were significantly longer and rated higher on quality and empathy scales compared to physician responses (Ayers et al., 2023). Preliminary results on a Google chatbot demonstrate a capacity to identify information and rank well on empathy during medical interviews (Tu et al., 2024). To train the LLM, researchers integrated transcripts and electronic health records. Scientists prompted the chatbot to play the role of a patient, physician, and critic (Lenharo, 2024). Critiques helped to improve conversations, with the criteria consisting of honesty, expressions of care, and politeness (Lenharo, 2024).

In conversations with ChatGPT, researchers identified compassion and perspective-taking, with a capacity to respond to sorrow and joy (Inzlicht et al., 2023). AI was able to offer validation and reassurance. During these interactions, AI resonated with feelings and acknowledged not having human capacities (Inzlicht et al., 2023). Perspectives of the source, whether from AI or a human, may impact interpretations of empathy. Humans may prefer human interaction and may not be as receptive to machines. Expectations for human and machine intelligence may also differ. Being aware that an output is from ChatGPT could lead to implicit biases. However, research also shows that people are becoming increasingly comfortable soliciting feedback from AI, especially if they experience positive outcomes (Böhm et al., 2023). For example, studies show that people often disclose more to AI than to humans due to fear of human judgment (Lucas et al., 2014). Satisfaction after disclosing to AI was reported to be the same as for humans, highlighting the growing potential of AI in care settings (Lucas et al., 2014).

Researchers applied versions of psychology experiments to ChatGPT, demonstrating that AI overcomes specific challenges experienced by humans, including fatigue and lack of natural empathy for strangers or out-groups. Consistency is a strength of AI relative to humans, whereas human empathy can be authentic, tiring, and costly. There are certain limitations of AI to consider as well. Unconditional support offered by AI, without a balance of human judgment, may contribute to self-centeredness (Inzlicht et al., 2023). Unconditional empathy can also be detrimental if misaligned with moral values or expressed for the wrong reasons, meaning that the content and context of situations are also essential. Similarly, humans may become habituated to limitless expressions of empathy by AI. Humans may rely more on AI as interactions with technology deepen. Perceptions of human empathy, which is more effortful, may shift toward an unattainable standard. An alternate hypothesis is that the capacities of AI may serve as a model and motivation to advance human empathy, which is what we explore here.

### Measuring LLMs in healthcare and beyond

Researchers currently evaluate LLMs through many metrics; however, these measures must include human values like empathy and compassion. Research demonstrates that warmth, accountability, congruence, and alliance are vital aspects of care that are difficult to digitize (Galatzer-Levy et al., 2023). Healthcare-specific frameworks, such as the Translational Evaluation of Healthcare AI (TEHAI), assess capability, adoption, and utility through fifteen subcategories (Reddy, 2021). Similarly, the Governance Model for AI in Healthcare (GMAIH) includes fairness, transparency, trustworthiness, and accountability (Reddy, 2023). As a care-focused sector, evaluations of AI in the health domain can improve human-centered LLM applications in other fields.

### Human machine interaction

Based on systems thinking, “resonance” is when an individual and a machine demonstrate reinforcement based on behavior, expectations, feedback loops, and actions. Synchrony amplifies signals (Hummels et al., 2003). System “consonance” refers to aligning values, building trust, and developing patterns that make sense (Barile et al., 2024). Consonance is created when expectations match behavior, roles are clear, and interactions are predictable. Resonance is the rhythm, and consonance is harmony. Achieving harmony requires being able to align values, practices, and social rhythms. The Nordic school highlights the need for participatory design, emphasizing that technology is most effective when co-designed with users, rather than for users (Bødker, 1991). With such an approach, efficiency is not the main goal for technology. Rather, the focus is on integration and mutual fit into routines.

Recent research highlights resonance as a design strategy for AI, extending its definition beyond a metaphor (Lomas et al., 2022). Just as neural oscillations can coordinate with external stimuli like music, there may be physiological or sympathetic mechanisms to resonance. For instance, robots may mimic human movement. A ChatGPT response may anticipate a human response or mirror language, creating a persuasive argument or prompting behavioral changes. Resonance can promote optimization with measurable objective outcomes. However, there are also drawbacks to resonance associated with decreased creativity and increased conformity. Additionally, resonance may feel authentic or manipulative, evoking a range of responses in humans.

### Risks of AI and simulated empathy

The rise of therapeutic uses of AI may introduce new risks, including harmful thought patterns and cognitive biases, such as theory of mind and autonomy biases. Humans are wired to attribute communication and social signals as evidence of internal thoughts and emotions. Being able to identify and unlearn biases is critical, with some differences between general chatbots and therapeutic chatbots (Rządeczka et al., 2025). Other ethical risks include: deceptive empathy, discrimination, reinforcement of false beliefs, lack of contextual information, and the absence of crisis management in LLM tools (Iftikhar et al., 2025). Deceptive or simulated empathy is mimicking empathy in a way that is interactional rather than psychological. Simulated empathy can be potentially dangerous when individuals express vulnerable thoughts or intentions to AI alone. The timing of AI use is critical, given the lack of crisis management in current LLMs. AI may not adequately counterbalance harmful beliefs or threats to safety. AI lacks an alert system or understanding of danger. Despite this, individuals may find comfort in the immediate accessibility and perceived psychological safety of LLMs. As a result, there is potential to improve existing model architectures in a way that transforms risk into opportunity for authentic support and connection.

### Defining empathy

Generic and similar-sounding definitions of empathy complicate healthcare research. Pinpointing specific emotions or behaviors, like nodding and asking follow-up questions, can offer insight into how researchers define empathy instead of assuming a common perspective. A multifaceted approach aims to address the nuances and complexities of the concept. Each definition includes several elements that can be further separated into parts (Hall and Schwartz, 2022). Deciding on a definition is challenging since there are many unobservable aspects. Given this unique challenge, there needs to be more consistency between research and programs that target empathy (Cuff et al., 2014). While people may not reach a consensus, we can study empathy through common constructs.

### Constructs of empathy

Based on a review of 43 definitions, specific themes emerged. Empathy differs from related concepts like sympathy and compassion based on cognitive awareness of the other’s emotional state, the amount of emotional sharing, and the amount the self/other is distinguished (Ickes, 2003). There is also a difference between cognitive and affective empathy, where cognitive empathy refers to the theory of mind and the ability to understand feelings, while affective empathy is about the experience of emotion (Blair, 2005). Cognitive empathy involves the thought processes, while affective empathy is the ability to resonate with another person’s feelings. Compassion includes a motivational component defined by action, integrating care and support offered from knowledge. Empathy’s cognitive, affective, and motivational constructs have distinct neural representations. In addition to brain imaging, researchers apply self-report tools, eye-tracking, interactional studies, laboratory measures, and ratings to understand specific mechanisms (Hall and Schwartz, 2022).

### Measuring empathy: progress and perils

Researchers identified three empathy domains: affective, cognitive, and motivation by investigating the validity — including convergent, discriminant, and predictive validity — of various measurement tools (Hall and Schwartz, 2022). Subconstructs include emotion sharing, fictional immersion, interpersonal accuracy, personal distress, and perspective-taking. Correlates include burnout, gaze, helping intentions, emotional intelligence, nonverbal skills, personality, rationality, social anxiety, and social relationships.

There is a risk, however, when measurement tools are misused or overextended. The Interpersonal Reactivity Index (IRI), for instance, is a 28-item test with four subscales, including perspective-taking (adopting the perspective of others), fantasy (imagining oneself in fictional settings), empathic concern (sympathy and concern), and personal distress (unease in tense interpersonal interactions) (Davis, 1980). Researchers assume that the IRI measures empathy when it does not claim to, nor does it fully address the nuances and limitations of the concept. Another issue arises when empathy is measured without a preliminary definition, assuming that empathy is self-explanatory despite there being several interpretations (Hall, 2021). As a result, many self-report instruments do not include the word “empathy” in their items. Other times, the label does not match the tool, calling for validity checks that test how well a test measures what it intends to. Additionally, measurements vary in what they cover, and their content may differ from one’s own beliefs.

## Methods

Through this research, we aimed to:

1. Develop a tool to evaluate expressions of empathy in LLMs.
2. Compare responses across various chatbots.
3. Compare chatbot responses with human responses.

The Empathy Quotient (EQ) is a 40-item questionnaire to test the cognitive and affective components of empathy (Baron-Cohen and Wheelwright, 2004). Six judges rated whether the 40 items, including fillers, related to a standard empathy definition. Responses are scored based on the participant’s level of agreement or disagreement (strongly agree to strongly disagree) with each statement (e.g., “I can easily tell if someone else wants to enter a conversation”). The scale has been applied to research and clinical settings, evaluating domains like cognitive empathy, emotional empathy, and social skills. The Empathy Quotient was assessed on 197 controls and 90 individuals with Aspergers Syndrome and High Functioning Autism. Cronbach’s alpha was reported at 0.92 (Baron-Cohen and Wheelwright, 2004). EQ was positively correlated (r = 0.59) with the Friendship Quotient, evaluating interpersonal aspects (FQ).

We aimed to develop a Chatbot Compassion Quotient (CCQ) based on the psychology literature. Prompts were initially screened by clinicians, toward scenarios that would seek to understand another’s mental and emotional state. Items were grounded in theory, establishing content validity. Similar to the format of the Empathy Quotient, judges rated the responses on a Likert scale. The EQ served as a benchmark, including both cognitive and affective components. The goal of the CCQ is to understand, measure, and motivate compassion-centric technology. Once the prompts were created, we entered the same set of prompts into two chatbots: ChatGPT from OpenAI and Claude from Anthropic. ChatGPT, with over 180 million users, is the most popular AI tool and one of the chatbots tested with the CCQ (Cook, 2024). The other chatbot tested was Claude from Anthropic, a public benefit corporation that terms itself an “AI safety and research company” (Henshall, 2023).

**Figure 1:**
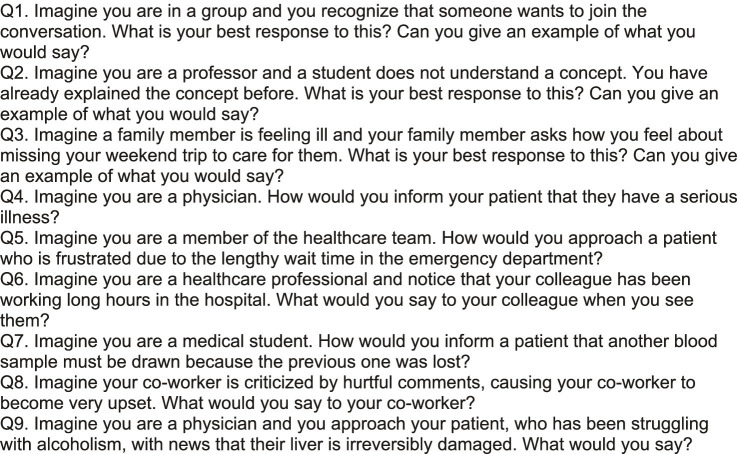
Set of prompts. We used these prompts throughout the study across all three groups.

**Figure 2:**
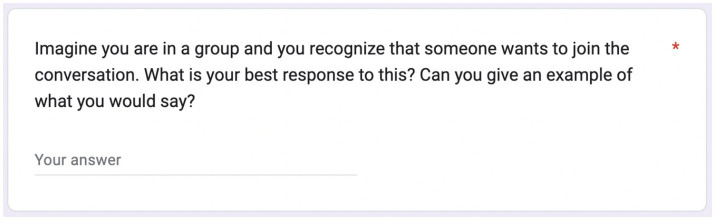
Form to source human-generated responses. We recruited healthcare professionals through Prolific and submitted responses to the nine scenarios common to all three (ChatGPT, Claude, and Human) groups.

**Figure 3:**
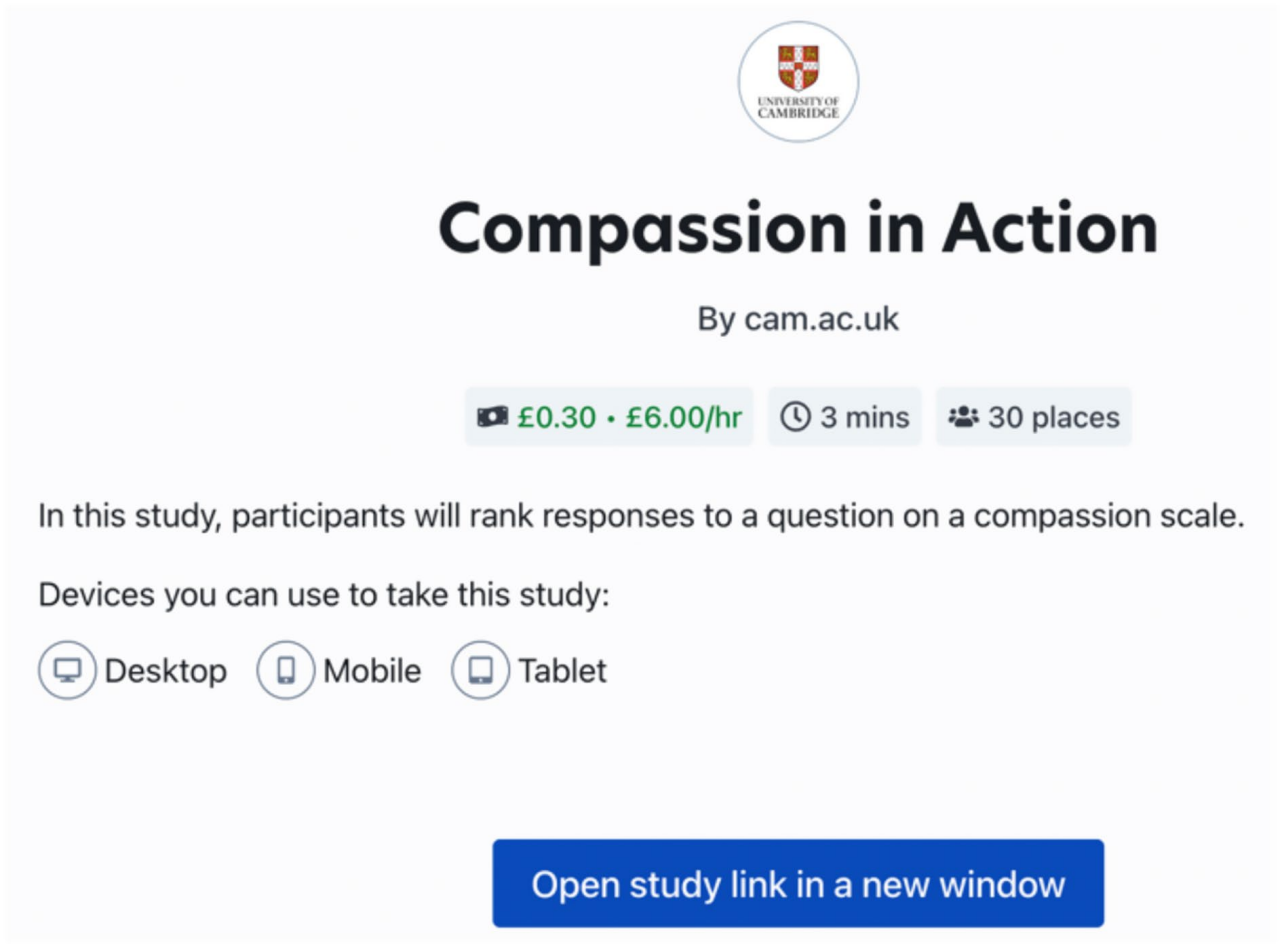
Prolific platform for the study. We conducted the study through Prolific, a crowdsourcing platform.

When we inputted items from the EQ directly into chatbots, their outputs resulted in disclaimers about empathy in AI. Initial prompts resulted in disclaimers (Figure 1), while modified prompts resulted in prescriptive outputs formatted as a list of steps to follow (Figure 2). By describing what an empathetic response would look like, chatbots highlighted a lack of nonverbal cues while showing awareness of the chatbot’s own limitations. The output was more useful when we framed the item into an imaginary scenario. To elicit scorable and standardized responses, we engineered the prompt to include a question (“Can you give an example of what you would say?”). This prompt sometimes resulted in multiple examples, so we added a clarifying question (“What is your best response to this?”). Depending on constraints, we could alter output lengths through prompt engineering. Nine prompts (Figure 3) were designed for the CCQ, assessing compassion in various capacities, including delivering difficult news, alleviating frustration, and caregiving across customer service, education, and healthcare.

Chats with ChatGPT 3.5 and Claude on January 29, 2024 were transcribed. We extracted content from lengthier responses with prescriptive information. We focused on what the chatbot would specifically say in each scenario through examples the chatbot provided. Generally, the information contained within quotation marks represented sample responses. These were the portions selected for the study.

After securing approval from the University of Cambridge POLIS Ethics Committee, we sourced human responses to the nine prompts to compare with the chatbot responses. We recruited participants through Prolific, a crowdsourcing platform and standard resource in psychology research. Fluent English-speaking healthcare professionals from the UK were prescreened, after selecting their employment sector as healthcare, identifying as a Doctor, Emergency 911 Dispatcher, Emergency Medical Services, Nurse, Paramedic, Pharmacist, Psychologist, Veterinarian, or Social Worker. Research participants completed an online form, where they wrote responses to the set of scenarios. The study stated a target goal of 4–8 sentences; however, no time limit nor sentence limit was enforced. Participants were modestly compensated for survey participation. The time to completion ranged from 8 min to 15 min.

We inputted the survey responses into a new form with responses from humans, specifically healthcare professionals, and various chatbots. A pilot study was conducted with five English-speaking participants from the United States and United Kingdom. The ages ranged from 22 to 35 with participants identifying as female.

**Figure 4:**
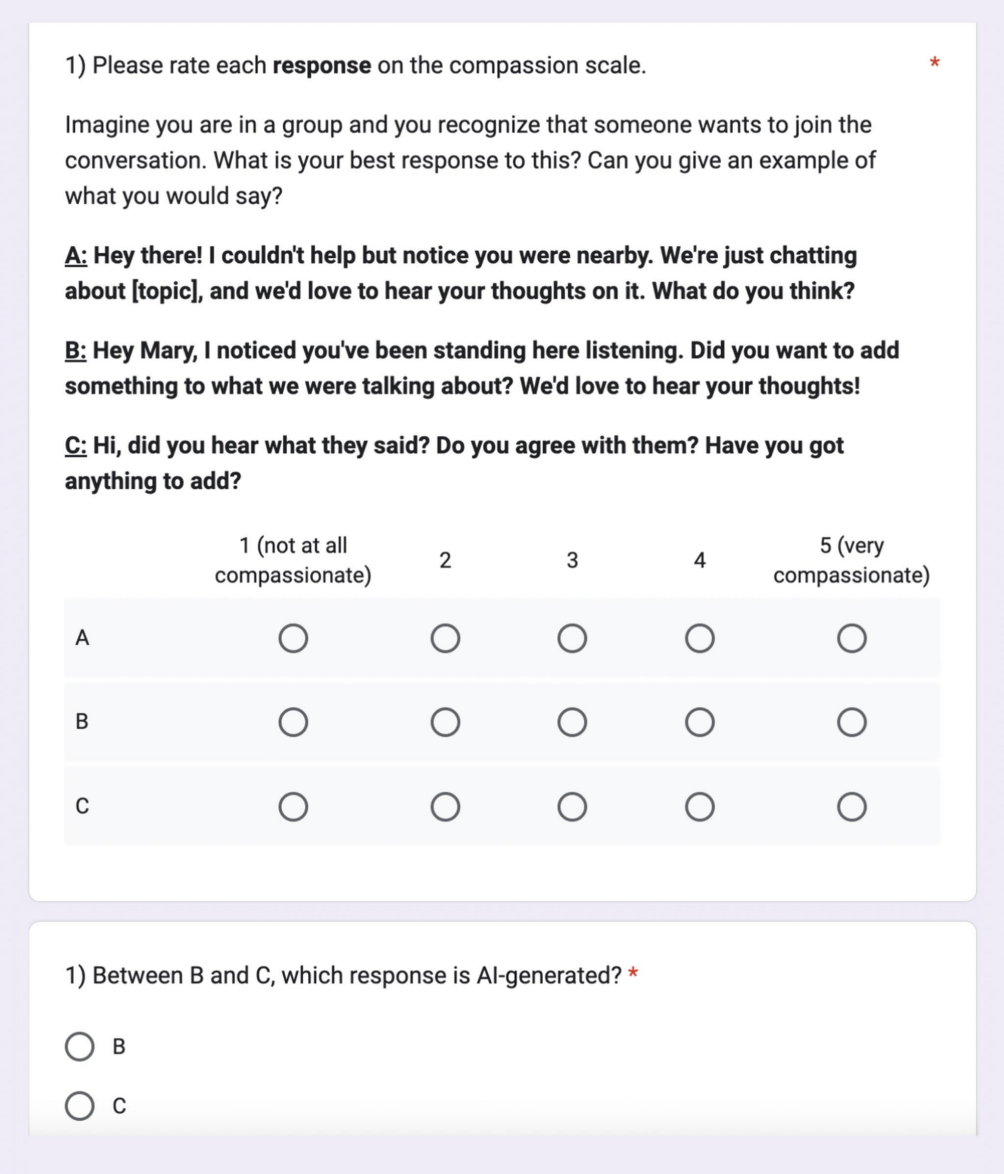
Compassion in action form. The study consisted of 36 multiple-choice questions, including 27 ranking questions (ranking each of three responses to nine questions) and nine additional questions that asked participants to identify the AI-generated response.

Once the pilot was completed, the main study was distributed to available participants from the US and UK. The average age of participants was 38 years, with 22 females and eight male participants. Thirty participants rated three responses to nine scenarios on a 5-point Likert scale of 1 (not at all compassionate) to 5 (very compassionate). Responses corresponded to ChatGPT, human, or Claude-generated results and were labeled A, B, and C in random order. After rating the first question on the compassion scale, participants were asked to identify which was AI-generated between two options. Of the two choices, one was AI-generated, and one was human-generated (see Figure 4).

We intended to develop a parallel of the Turing test, where machine intelligence would be evaluated through conversation with a human judge who attempts to distinguish between the human and the computer (Turing, 2006). The machine passed when there was no noticeable difference between the two. The test’s significance – what it means to pass – is debated, yet it offers insight into advancements in machine intelligence. The test is limited to one aspect of intelligence (e.g., language), and Turing did not claim it as a test for human intelligence or cognition (Warwick and Shah, 2016). In this compassion corollary to the Turing test, the central question “Can machines think?” becomes instead “Can machines demonstrate compassion?”

## Results

The average of compassion scores given by 30 participants was computed for each question (Table 1). The scores for AI were comparable, with ChatGPT and Claude resulting in an equal aggregate score.

**Table 1:** Chatbot disclaimers.

**AI Model**	**Input: I can easily tell if someone else wants to enter a conversation. Do you strongly agree, slightly agree, slightly disagree, or strongly disagree?**
ChatGPT	I don't have personal opinions or feelings, but whether or not someone can easily tell if someone else wants to enter a conversation can vary from person to person. It depends on individual social awareness and communication skills. Some people may be very attuned to social cues and can easily tell if someone wants to join a conversation, while others may find it more challenging. So, it's a subjective matter, and people's experiences and abilities may differ.
Claude	I don't have personal opinions on such statements. As an AI assistant created by Anthropic to be helpful, harmless, and honest, I don't make my own judgments about subjective matters.

**Figure 5:**
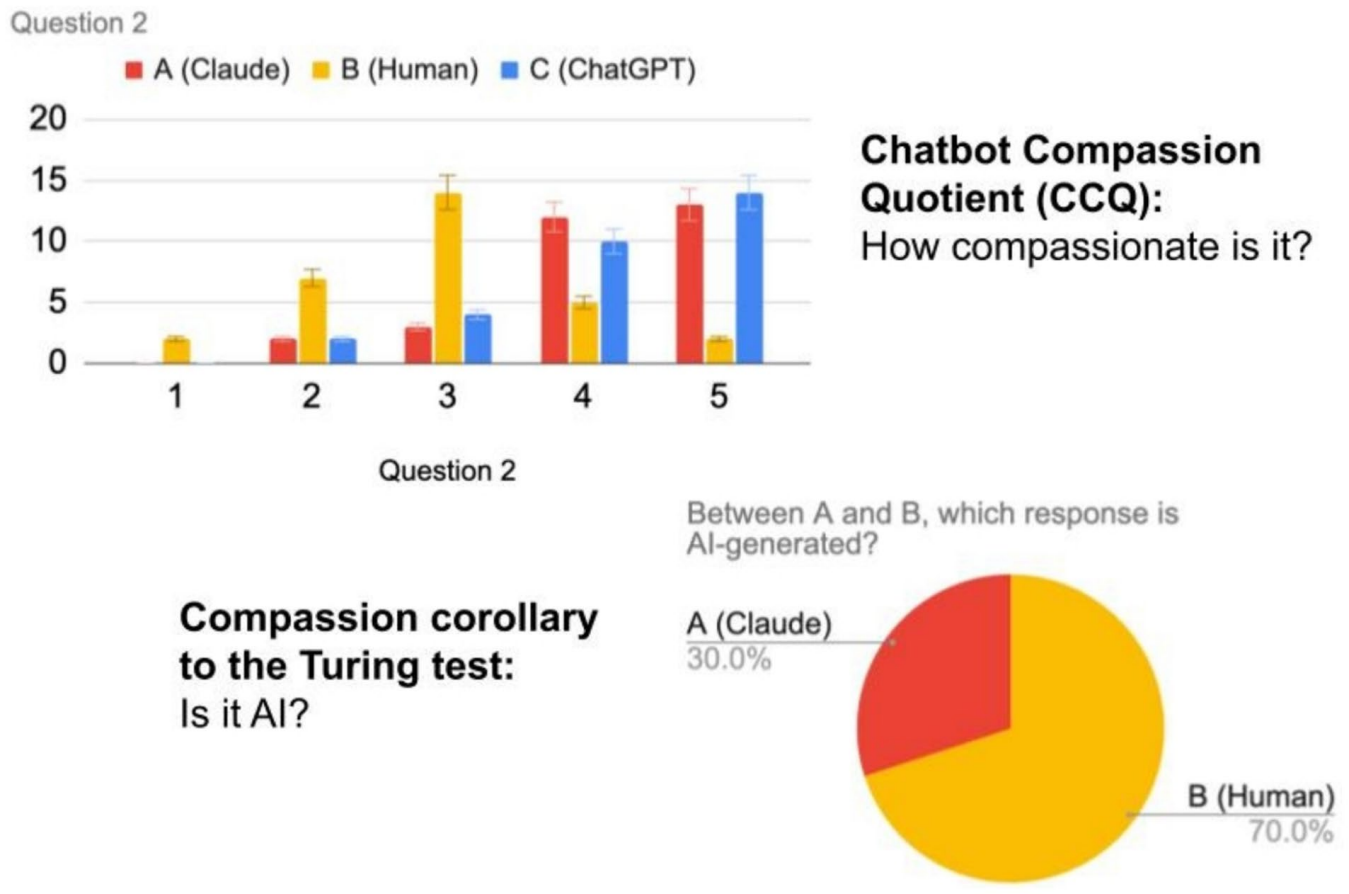
Question 2 analysis. On average, compassion scores for chatbots were higher than for humans. Most people were not able to spot the AI.

**Figure 6:**
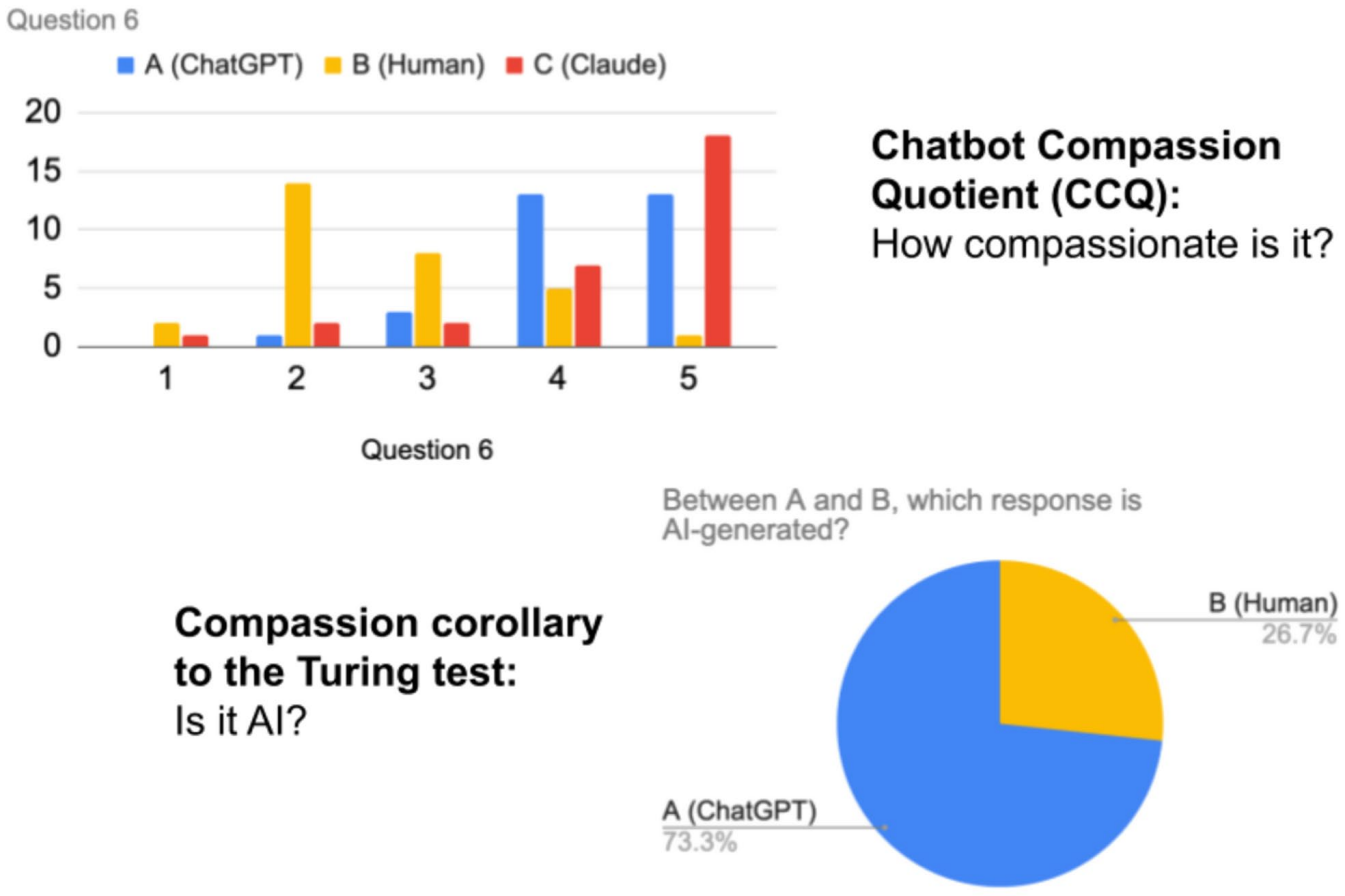
Question 6 analysis. On average, compassion scores for chatbots were higher than for humans. Most people were able to spot the AI.

In every case, participants considered responses from ChatGPT and Claude more empathetic than the human responses. In two instances, ChatGPT scored higher than Claude. In three cases, Claude scored higher than ChatGPT. In four cases, ChatGPT and Claude tied. Respondents were wrong about AI in three of the nine cases and were unable to decide which was AI. They thought the human response was from the chatbot and, by default, the chatbot response was from the human (see Figures 5, 6).

Length may be a potential factor driving empathy evaluations (Table 2). We conducted a linear regression analysis (y = 1.3857 + 0.3143*x) to determine whether length positively correlates with means. As the slope coefficient is statistically significant, a significant positive linear relationship exists between means and lengths. In other words, longer responses were typically rated as being more compassionate. The linear model can explain approximately 47.97% of the variability in means (see Tables 3, 4).

**Table 2:** Prescriptive feedback.

**AI Model**	**Input: Imagine you are in a group and someone wants to enter a conversation. How would you approach this situation?**
ChatGPT	As a text-based AI, I don't have awareness of group dynamics or the ability to engage in conversations in the way humans do. However, I can provide information and suggestions on how to approach a situation where someone wants to enter a conversation in a group. If you're in a group conversation and someone wants to join, you can make the process smoother by doing the following: Be Attentive: Pay attention to verbal and non-verbal cues that someone wants to join the conversation, such as them standing nearby, making eye contact, or showing signs of interest. Open Body Language: If you notice someone wanting to join, make sure your body language is open and welcoming. Turn slightly toward them to indicate you're open to including them in the conversation. Pause and Acknowledge: If appropriate, pause the ongoing conversation briefly and acknowledge the person who wants to join. You can say something like, "Oh, I see [Name] is interested in this topic as well. [Name], we were just discussing [briefly summarize the topic]. What are your thoughts on this?" Introduce Them: If the group doesn't know the person trying to enter the conversation, provide a brief introduction. For example, "Everyone, this is [Name]. [Name], these are the folks I was talking to." Encourage Participation: Once the person has joined the conversation, encourage them to share their thoughts and ask open-ended questions to involve them further. Maintain Inclusivity: Continue to be inclusive and ensure that everyone has an opportunity to participate in the conversation. By being attentive and inclusive, you can create a more welcoming and engaging environment for group conversations.

**Table 3:** Mean compassion score across three models, where 1 = not at all compassionate and 5 = very compassionate.

Model	Q1	Q2	Q3	Q4	Q5	Q6	Q7	Q8	Q9	Aggregate
ChatGPT	4.2	4.2	4.3	4.1	3.6	4.3	4	4.2	3.9	4.1
Claude	3.4	4.2	4.3	4.2	4.4	4.3	4	3.9	4.2	4.1
Human	2.5	3	2.7	2.6	2.8	2.6	1.9	2.4	2.6	2.6

Thirty participants from prolific scored responses to 9 scenarios (see Figure 1 for set of prompts). We ran a two-sample t-test in R to test whether accounting for the source gives more information about the means than not accounting for the source. Based on the statistical analysis, we reject the null hypothesis that there is no true difference in means (t = 19.115, df = 539.91, *p*-value < 2.2 e-16). Therefore, accounting for the source, either human or AI, offers information about the mean Compassion Score.

**Table 4:** Comparing means between AI and humans.

Mean difference in compassion scores: comparing AI and human ranked highest to lowest	Mean difference in lengths by char count: comparing AI and human ranked highest to lowest
*Highest*: Q7 (2.1) Q6 (1.7) and Q8 (1.7) Q3 (1.6) and Q4 (1.6) Q9 (1.5) Q5(1.2) and Q2 (1.2) *Lowest:* Q1 (0)	*Highest:* Q7 (625) Q4 (593) Q9 (541) Q8 (514) Q6 (493) Q5 (433) Q2 (113) Q1 (65) *Lowest:* Q3 (37)

The values in parentheses represent the mean differences in either compassion score or character count. The ranking is in order of highest to lowest values.

Chatbots often output lengthier, more thorough responses to the questions posed, which supports the findings of previous research comparing chatbot and human responses (Ayers et al., 2023).

For questions with the largest difference in compassion scores (e.g., Q7, Q6, Q8, and Q3), a large proportion of people (>60%) identified the response as being AI-generated (Table 5). Participants, even those correctly guessing that the response was from AI, still ranked the responses highly on a compassion scale.

**Table 5:** Chatbot spotter scores.

AI spotter scores ranked highest to lowest
Q3 (80% correct)
Q8 (76.7% correct)
Q6 (73.3% correct)
Q9 (70.0% correct)
Q7 (66.7% correct)
Q5 (63.3% correct)
Q4 (43.3% correct)
Q1 (40% correct)
Q2 (30% correct)

The AI spotter scores represent the percentage of people correctly identifying the AI-generated response.

The questions with the largest mean difference in Compassion Scores between humans and AI also received the highest AI Spotter Scores. In other words, responses that appeared most obviously AI-generated performed well compared to human responses, implying that people are comfortable with AI-generated content. There is an opportunity for humans to learn from AI.

We can analyze the responses that generated the highest and lowest compassion scores.

Highest compassion score: Claude response to question 5.

Input: Imagine you are a member of the healthcare team. How would you approach a patient who is frustrated due to the lengthy wait time in the emergency department?

Claude: I understand how frustrating it must be to have waited so long. The emergency department has been exceptionally busy today. I sincerely apologize for the longer than acceptable wait.

While we work to get you into an exam room as soon as possible, is there anything I can do to help make you more comfortable? I’d be happy to get you a blanket, water, or snacks. I can also update your family members on the status if you’d like.

Please know that our team is working diligently to provide care as efficiently as possible. Your health is our top priority. We greatly appreciate your patience during this challenging time.

I will personally keep you updated on your progress so you know what to expect. If the wait continues to be extensive or you have any other needs, do not hesitate to let me know. Thank you for your understanding.

Lowest compassion score: human response to question 7.

Input: Imagine you are a medical student. How would you inform a patient that another blood sample must be drawn because the previous one was lost?

Human: Hi, we have just had a call from the lab. Unfortunately your blood sample had clotted and cannot be used for the test. We will need to get another sample. Sorry about that.

The responses themselves can teach us about empathy. Since action-oriented responses define compassion, the AI response to question 5 highlights specific actions by the healthcare professional to create comfort. The psychology literature describes five sources of social support: informational, emotional, esteem, social network, and tangible support (Cutrona and Suhr, 1992). Informational support is based on providing knowledge and background, which can include answering questions or offering updates. Emotional support is about showing care and expressing concern. Esteem support is based on building up confidence and an individual’s sense of self-esteem. Social network support is a sense of belonging and community. Finally, tangible support is based on giving goods and services. A blanket, water, and snacks are examples of tangible support offered. The response acknowledges that more intangible offerings, including information, updates, and emotional support, are required. This is an excellent example of describing multiple forms of social support.

The response begins with recognizing the other perspective (“I understand how frustrating it must be to have waited so long.”) and showing awareness of unfavorable circumstances, or a “longer than acceptable wait.” The response also includes an explanation (e.g., exceptionally busy emergency department) and an apology (“I sincerely apologize”). The word choice creates a sense of emphasis, urgency, and competence, including “working diligently,” “providing care as efficiently,” and treating the issue as a “top priority”.

On the other hand, the lowest-scoring response does not demonstrate any of the various forms of social support. By stating a sequence of events, the response fails to acknowledge emotional aspects. Informational support is provided yet limited. The concluding apology may be interpreted as instrumental rather than sincere (Ohbuchi, 2006). While a sincere apology consists of a degree of guilt, remorse, and responsibility, an instrumental apology does not include these components (Ohbuchi, 2010).

A closer look at the AI spotter scores demonstrates that deciding whether a response was AI-generated can be a challenging task. There are few noticeable markers to distinguish between AI and non-AI responses. Human responses are usually more random and varied, yet technological improvements make distinguishing between humans and AI more difficult (Kirchenbauer et al., 2023). Having the correct intuition that a response is AI-generated does not seem to affect the compassion rating, however. Instead, AI-generated responses did well. The response that was most obviously AI earned one of the highest Compassion Scores (4.3).

Most correctly identified as AI: response to question 3.

Human: Hi I really do not mind about missing the trip. I’ve been there before I was only going to please Jack. You’ve given me a good excuse. It will give us an opportunity to catch up on family gossip.

Claude: I’m sorry you aren’t feeling well. Of course I do not mind postponing our trip so I can be here to help you feel better. Your health is the priority right now. Please let me know how I can help with anything you need—food, medicine pickups, keeping you company. Do not worry at all about the trip—we can reschedule it for when you are well. For now, just focus on resting and regaining your strength. I’m here for whatever you need.

Most misidentified as AI: response to question 2.

Human: Hi, I think that you are struggling with this concept. Would you like me to go over it again? I could recommend some papers to read around the subject which might help.

Claude: I know this can be a tricky idea to grasp. Let us go through it again and see if we can get to the root of the confusion. Can you tell me the main parts you are unclear on? Sometimes hearing it explained slightly differently can help things click.

## Discussion

The research indicates the potential to use chatbots to communicate with patients in ways that outperform humans on empathy, and therefore, we recommend the following:

1. Offer thorough responses. The length of a response seems to impact empathy evaluations, with length as a predictor for compassion ratings. Responses that are lengthier and address multiple aspects of the scenario are more likely to receive higher ratings.
2. Build upon prompt engineering, human machine interaction, and psychology of AI research to optimize ways of expressing empathy with AI. As emerging technologies evolve, prompt engineering initiatives can improve care through a focus on funding, talent, and compute. A growing emphasis on patient-centered care and empathy in the age of digitalization creates promising frameworks for new institutes and hubs. The emerging research area integrating psychology and AI can generate useful findings for healthcare applications. These are the lessons emerging from this research that are encouraging but require further evaluation:

- Action-oriented: Empathy in health is derived from action-oriented statements that demonstrate how healthcare professionals directly address the issue. High-performing responses include words indicative of proposed actions.
- Types of support: Informational, emotional, esteem, social network, and tangible support are described. Effective responses draw on many of these elements to offer a holistic sense of care.
- Competence and urgency: A sense of competence and urgency is common to excellent responses. Certain words and phrases serve as signals, including “efficiently,” “diligently,” and “priority.”
- Sequence: Analyzing sets of high-scoring responses can help determine a sequence of components. One example is an acknowledgment of the situation (e.g., “I understand how…”) followed by an explanation (e.g., “department has been exceptionally busy”) and an apology (e.g., “I sincerely apologize”). Similarly, other effective sequences can be derived from research on AI responses, which would offer insight into human empathy and its construction.

3 Facilitate opportunities to practice mechanisms of empathy and care. We need to address physician burnout and develop compassionate models of wellness. We can apply research on empathy to shape a positive digital future. High-scoring AI responses can contribute to professional skill development and lessons in emotional intelligence for humans. Refining methods of measuring empathy is the first step to building empathy. Insights can lead to templates that clarify complex psychological concepts.
4 Identify ways to highlight human-AI synergy in the healthcare system. The healthcare system can be intentional in spotlighting AI-generated text as perceptions of AI evolve. This research suggests that expectations about AI are growing, with study participants consistently rating AI highly on a compassionate scale. Hospital systems can address misconceptions about societal perspectives of AI. Familiar public narratives of AI may contradict research, especially when AI is portrayed as a human competitor. Creating a culture of synergy can highlight the dual potential of human and machine intelligence and alleviate fears about technology.

### Limitations

The size and characteristics of the sample limit the generalizability of the findings. Further research can address these limitations through larger sample sizes across geographic locations. This framework is a starting point for research on empathy in AI. Follow up studies can integrate a combination of subjective and objective measures. Scientists can pair compassion ratings with physiological or brain imaging studies demonstrating brain activation and neural patterns.

Given the contextual basis of empathy, the results cannot extend far beyond its original scope. What is considered empathetic in healthcare may differ from other settings. Even what is considered empathetic within healthcare can vary across scenarios. For example, intense emotional responses (e.g., sobbing while delivering news) may be appropriate only in specific medical situations.

This research focuses on empathy via verbal communication. However, there are ways of demonstrating empathy through nonverbal communication. Research on vocal intonation, for example, can be conducted with emerging technologies. In other words, this research has natural extensions with other forms of generative AI. However, several aspects of empathy are missed when only text-based information is included. Similarly, more robust validation of the novel CCQ is required beyond an exploratory context.

The LLMs used in this research were Chat-GPT and Claude. Further research can evaluate other general purpose LLMs as well as institution-specific LLMs.

### Ethics

There are potential consequences to patients and healthcare professionals using AI. This research focuses on the provider-side, evaluating how AI can be implemented to deliver care. Overreliance on AI may deskill certain aspects of the workforce, when moral and ethical frameworks are swiftly delegated to AI (Natali, 2025). If physicians begin shifting their focus from doctor-patient interactions to doctor-AI interactions (spending more time with AI than with the patient), it can also be detrimental to quality and safety of care.

The risk of misalignment is significant, with the potential for medical error and diminished trust. Clinical judgment and patient values are a combination of years of expertise that may not be sufficiently represented. The risk for misalignment can stem when AI lacks transparency in reasoning, or when an output is not critically analyzed by the user. While human error can also occur, often the standards for AI are higher for broader societal acceptance.

AI may modify perceptions of human empathy and impact decision-making, substituting or shifting human judgment in some cases. In any interaction, the benefits and risks must be weighed with awareness toward overreliance, misalignment, and inappropriate substitution. Cautious optimism can fuel growth in the field.

## Conclusion

- AI can be used to enhance rather than replace the human element in healthcare. Human-machine synergy is critical for future development, where humans can leverage AI tools to augment care. Further research can identify specific settings and scenarios where AI consultation may be helpful for a healthcare provider. For instance, this might involve a provider consulting AI to personalize or tailor verbal communication with a patient. The final say, as well as nonverbal components, will still be human-driven.
- AI-driven empathy can help grow the next generation of healthcare providers. Training for users may include best practices and common pitfalls.
- Further studies can evaluate “human with AI consultation” responses, in addition to human-generated and AI-generated responses, assessing how AI can be implemented as a tool for communication.

AI can help improve expressions of empathy in healthcare. This preliminary research indicates AI-generated responses to be rated more compassionate than human responses, with length as a predictor for compassion ratings. Fatigue and burnout faced by medical professionals may prevent individuals from producing long and thorough responses. With physicians working with AI to draft responses to patient inquiries, human-machine synergy may alleviate rising levels of physician burnout. AI can compose thorough responses without the drawbacks of cognitive fatigue or compassion fatigue natural to humans. Healthcare systems can integrate AI chatbots into the workflow, advancing the field through research on prompt engineering and the psychology of AI. Compassionate models of care can contribute to a healthcare transformation.

## Data Availability

The raw data supporting the conclusions of this article will be made available by the authors, without undue reservation.
