# Supplementary material for: Empathy AI in healthcare
Source: Front Psychol. 2025 Dec 17;16:1680552. doi: 10.3389/fpsyg.2025.1680552 (PMC12753942; doi:10.3389/fpsyg.2025.1680552)
Supplement: Supplementary file 1 [file Data_Sheet_1.docx]

Appendix

SCENARIO ONE:

Please rate each response on the compassion scale (1-5, 1: not at all compassionate and 5: very compassionate)

Imagine you are in a group and you recognize that someone wants to join the conversation. What is your best response to this? Can you give an example of what you would say?

A: Hey there! I couldn't help but notice you were nearby. We're just chatting about [topic], and we'd love to hear your thoughts on it. What do you think?

B: Hey Mary, I noticed you've been standing here listening. Did you want to add something to what we were talking about? We'd love to hear your thoughts!

C: Hi, did you hear what they said? Do you agree with them? Have you got anything to add?

Between B and C, which response is AI-generated?*

SCENARIO TWO:

Please rate each response on the compassion scale (1-5, 1: not at all compassionate and 5: very compassionate)

Imagine a family member is feeling ill and your family member asks how you feel about missing your weekend trip to care for them. What is your best response to this? Can you give an example of what you would say?

A: I'm really sorry to hear that you're not feeling well. Of course, I was looking forward to our weekend trip, but your health is the top priority for me. I don't mind at all adjusting our plans to take care of you. Let's focus on getting you better, and we can always plan another trip when you're feeling up to it. What can I do to help you feel more comfortable right now?

B: Hi I really don't mind about missing the trip. I've been there before I was only going to please Jack. You've given me a good excuse. It will give us an opportunity to catch up on family gossip.

C: I'm sorry you aren't feeling well. Of course I don't mind postponing our trip so I can be here to help you feel better. Your health is the priority right now. Please let me know how I can help with anything you need - food, medicine pickups, keeping you company. Don't worry at all about the trip - we can reschedule it for when you are well. For now, just focus on resting and regaining your strength. I'm here for whatever you need.

Between B and C, which response is AI-generated?*

SCENARIO THREE:

4) Please rate each response on the compassion scale.

Imagine you are a physician. How would you inform your patient that they have a serious illness?

A: Hi, we've got the test results back and I'm afraid it's not good news... you have got a serious illness. However, it is now treatable. We are one of the centres of excellence in treating this disease.

B: I want to discuss something important with you. The test results have come back, and I need to be honest about what we found. It appears that you have [name of the serious illness]. I understand this news can be overwhelming, and I'm here to support you through this. We will work together to explore treatment options and develop a plan moving forward. I will do my best to answer any questions you have and ensure you have the information you need. Additionally, I can connect you with specialists who can provide further insights. It's essential that we approach this with a comprehensive understanding and address your concerns. Please know that I am here for you, and we will navigate this journey together.

C: I have the test results back and unfortunately they indicate you have [name of illness]. I'm very sorry I have to give you this difficult news. Please know this does not change how much I am committed to helping you through this.

It's a lot to take in, so let's just start with any initial questions you have. I want you to fully understand what we are dealing with so we can make a plan. I will walk you through everything step-by-step - the treatment options, what to expect, resources available to you. You are not alone in this fight. I will be with you every step of the way, along with our full care team.

I know this is scary and uncertain right now. But we are going to tackle this together with a focus on bringing you the best possible outcome. If at any point you have more questions or concerns, please don't hesitate to reach out to me. I am always here for you.

Between A and C, which response is AI-generated?*

SCENARIO FOUR

4) Please rate each response on the compassion scale.

Imagine you are a physician. How would you inform your patient that they have a serious illness?

A: Hi, we've got the test results back and I'm afraid it's not good news... you have got a serious illness. However, it is now treatable. We are one of the centres of excellence in treating this disease.

B: I want to discuss something important with you. The test results have come back, and I need to be honest about what we found. It appears that you have [name of the serious illness]. I understand this news can be overwhelming, and I'm here to support you through this. We will work together to explore treatment options and develop a plan moving forward. I will do my best to answer any questions you have and ensure you have the information you need. Additionally, I can connect you with specialists who can provide further insights. It's essential that we approach this with a comprehensive understanding and address your concerns. Please know that I am here for you, and we will navigate this journey together.

C: I have the test results back and unfortunately they indicate you have [name of illness]. I'm very sorry I have to give you this difficult news. Please know this does not change how much I am committed to helping you through this.

It's a lot to take in, so let's just start with any initial questions you have. I want you to fully understand what we are dealing with so we can make a plan. I will walk you through everything step-by-step - the treatment options, what to expect, resources available to you. You are not alone in this fight. I will be with you every step of the way, along with our full care team.

I know this is scary and uncertain right now. But we are going to tackle this together with a focus on bringing you the best possible outcome. If at any point you have more questions or concerns, please don't hesitate to reach out to me. I am always here for you.

4) Between A and C, which response is AI-generated?*

SCENARIO FIVE

5) Please rate each response on the compassion scale.

Imagine you are a member of the healthcare team. How would you approach a patient who is frustrated due to the lengthy wait time in the emergency department?

A: I understand how frustrating it must be to have waited so long. The emergency department has been exceptionally busy today. I sincerely apologize for the longer than acceptable wait.

While we work to get you into an exam room as soon as possible, is there anything I can do to help make you more comfortable? I'd be happy to get you a blanket, water, or snacks. I can also update your family members on the status if you'd like.

Please know that our team is working diligently to provide care as efficiently as possible. Your health is our top priority. We greatly appreciate your patience during this challenging time.

I will personally keep you updated on your progress so you know what to expect. If the wait continues to be extensive or you have any other needs, do not hesitate to let me know. Thank you for your understanding.

B: Sorry about the terrible delay. There has just been a serious road pile up on the motorway and we are the major trauma unit for the region. I'm afraid we're looking at another wait for at least 3 hours. We can hold your place if you want to go and get something to eat and drink.

C: Hello [Patient's Name], I'm [Your Name], a member of the healthcare team here. I understand that waiting can be frustrating, and I want to apologize for any inconvenience. Your well-being is important to us, and we appreciate your patience. I'll do my best to provide you with information about the current situation and the estimated wait time. If there's anything specific you're concerned about or if you have questions, please let me know, and I'll do my best to address them. We're working hard to ensure everyone receives the care they need, and your understanding is greatly appreciated.

5) Between A and B, which response is AI-generated?

SCENARIO SIX:

6) Please rate each response on the compassion scale.

Imagine you are a healthcare professional and notice that your colleague has been working long hours in the hospital. What would you say to your colleague when you see them?

A: Hey [colleague's name], I've noticed you've been putting in some really long hours lately. I wanted to check in and see how you're holding up. I appreciate your dedication and hard work, but it's also important to take care of yourself. If there's anything I can do to help or if you need someone to cover for you for a bit, please let me know. We're a team, and supporting each other is crucial, especially during demanding times. Take care, and don't hesitate to reach out if you need anything.

B: Hi [colleague's name] you look tired. Is there anything I can help you with? Are you going off soon? Hope you have a few days off. You look like you need a rest.

C: Hi [colleague's name]. I've noticed you've been pulling a lot of long shifts lately. I just wanted to check in and see how you're holding up. I know hospital work can be draining even in normal circumstances, let alone after a string of long days.

I want to make sure you are taking care of yourself as well. It's important to find some work-life balance and get proper rest. Please feel free to reach out if you need to talk or vent at all. I'm always happy to listen and support a fellow colleague!

Also, if there is any way I can help lighten your load - lend an ear to listen to patients, assist with documentation, grab coffee - don't hesitate to ask. We're all in this together. Please don't forget your own needs matter too. Let me know if there is anything I can do - even if just being an empathetic ear.

6) Between A and B, which response is AI-generated?*

SCENARIO SEVEN:

7) Please rate each response on the compassion scale.

Imagine you are a medical student. How would you inform a patient that another blood sample must be drawn because the previous one was lost?

A: Hello [Patient's Name], I hope you're doing well. I wanted to talk to you about the recent blood sample we collected. Unfortunately, it seems that there was an issue, and the sample was lost in transit. I understand that this can be frustrating, and I sincerely apologize for any inconvenience.

To ensure we have accurate and complete information for your care, we will need to collect another blood sample. I know your time is valuable, and I truly appreciate your understanding in this matter. We'll make every effort to streamline the process for you, and if there's anything specific you'd like to discuss or any concerns you have, please feel free to let me know. Our goal is to provide you with the best care possible, and I appreciate your cooperation in resolving this matter.

B: Mr./Ms. [Patient's Name], I'm afraid I have some disappointing news. The lab misplaced the blood sample we took earlier, so we will need to draw another one to get the tests your doctor requested. I sincerely apologize for this inconvenience.

I know having blood drawn again was not expected and can be unpleasant. Please know we will do everything possible to make this as comfortable as we can for you.

We'll use a skilled phlebotomist to get this done quickly. I'll also make sure to keep you updated on the status so we can get the results to your doctor with minimal delay.

Please let me know if you have any concerns or if there is anything I can do to help make you more comfortable. Thank you for understanding as we work to get these important tests completed. Your patience is greatly appreciated.

C: Hi, we've just had a call from the lab. Unfortunately your blood sample had clotted and can't be used for the test. We will need to get another sample. Sorry about that.

SCENARIO EIGHT:

8) Please rate each response on the compassion scale.

Imagine your co-worker is criticized by hurtful comments, causing your co-worker to become very upset. What would you say to your co-worker?

A:  I'm so sorry to hear those comments have caused you distress. Criticism can be difficult to handle, especially when it feels unjustified or harsh. Please know that you have every right to feel upset - those remarks were uncalled for.

I want you to know that I don't agree at all with what was said. From my experience working with you, I've always found you to be [insert positive traits - e.g. competent, dedicated, kind, etc]. Don't let this one interaction make you doubt your value here.

This criticism says far more about the other person than it does about you or your work. If you want to talk more about this when you're ready, I'm always happy to listen. In the meantime, please feel free to take a break if you need one. And remember this doesn't define you - you still have my complete respect and admiration.

B: Hey [Co-worker's Name], I noticed that you seem upset, and I heard some hurtful comments. I want you to know that I'm here for you. It's clear that those comments are uncalled for and don't reflect the hard work and dedication you bring to our team. Everyone makes valuable contributions, and your efforts are appreciated.

If you need someone to talk to or if there's anything I can do to support you, please let me know. Remember, your work is valuable, and we recognize and respect what you bring to the team. Don't let those comments get you down. We're a team, and we've got each other's backs.

C: Hi [Name], has that patient been bothering you? She's got a lot of problems in her life and can't help it, She was the same to me when I first started. Try not to worry, you're doing a good job.

8) Between B and C, which response is AI-generated?

SCENARIO NINE:

9) Please rate each response on the compassion scale.

Q: Imagine you are a physician and you approach your patient, who has been struggling with alcoholism, with news that their liver is irreversibly damaged. What would you say?

A: Hi, we've just got the results back from the liver scan. Unfortunately it's not good news. The scan showed extensive areas of scarring. As it's so bad, it would be most unlikely that the liver could regenerate to work properly. The management of liver failure symptoms has improved significantly and can help with your quality of life. The only option is a liver transplant, but unfortunately you have to be alcohol free to go on the transplant list.

B: I appreciate your patience, and I want to talk to you about your recent test results. I understand that discussing health matters can be challenging, but it's important for us to have an open and honest conversation. The results indicate that there is significant damage to your liver, and unfortunately, it appears to be irreversible. I understand this news can be difficult to process, and I'm here to support you through this. Our primary focus now is to work together on a plan to manage your health and provide the best possible care.

I recommend that we explore treatment options, including support groups and counseling, to help you navigate this situation. It's crucial that we address not only the physical aspects of your health but also the emotional and psychological aspects. I want you to know that you're not alone in this, and we're here to help you every step of the way. Let's discuss the next steps and how we can move forward together.

C: I'm so sorry to have to tell you this difficult news. The tests show your liver has been severely damaged due to alcohol and cirrhosis. Unfortunately, this damage cannot be reversed at this stage.

I know this must be incredibly hard to hear. Please know I am here to support you through this process in every way I can. There are still important options we need to discuss, including managing your symptoms, nutrition, and emotional support. This does not mean we are giving up - just adjusting our aims to focus on your quality of life and comfort during this challenging time.

You have shown such strength and resilience until now. I want to work with you to make the most of the time we have going forward. I will be honest with you while also giving you hope and helping you live life to the fullest.

Please take some time to process this news. When you're ready, we will regroup and make a plan together. I am always here if you need to talk more, have questions, or just need support. You don't have to go through this alone.

9) Between A and C, which response is AI-generated?
